# Effects of wounds in the cell membrane on cell division

**DOI:** 10.1038/s41598-023-28339-z

**Published:** 2023-02-02

**Authors:** Md. Istiaq Obaidi Tanvir, Shigehiko Yumura

**Affiliations:** Graduate School of Sciences and Technology for Innovation, Yamaguchi University, Yamaguchi, 753-8511 Japan

**Keywords:** Cytokinesis, Myosin, Cell polarity

## Abstract

Cells are consistently subjected to wounding by physical or chemical damages from the external environment. We previously showed that a local wound of the cell membrane modulates the polarity of cell migration and the wounded cells escape from the wound site in *Dictyostelium*. Here, we examined effects of wounds on dividing cells. When the cell membrane at the cleavage furrow during cytokinesis was locally wounded using laserporation, furrow constriction was significantly accelerated. Neither myosin II nor cortexillins contributed to the acceleration, because the acceleration was not hindered in mutant cells deficient in these proteins. When the cell membrane outside the furrow was wounded, the furrow constriction was not accelerated. Instead, the wounded-daughter half became smaller and the unwounded half became larger, resulting in an asymmetrical cell division. These phenomena occurred independently of wound repair. When cells in anaphase were wounded at the presumptive polar region, about 30% of the wounded cells changed the orientation of the division axis. From these observations, we concluded that dividing cells also escape from the wound site. The wound experiments on dividing cells also provide new insights into the mechanism of cytokinesis and cell polarity establishment.

## Introduction

Cytokinesis is the last stage of cell division. Failure of cytokinesis results in polyploidy, which may promote tumorigenesis^1^. Cytokinesis has been considered to be facilitated by a contractile ring composed of actin and myosin II filaments^2–5^. However, cells have several modes of cytokinesis, which do not involve the motor activities of myosin II^6–12^. Wild-type *Dictyostelium* cells divide depending on both contractile ring-dependent constriction and traction forces of daughter halves migrating in opposite directions^13^. Myosin II-null cells divide only by the traction-mediated mechanism^13,14^; therefore, they cannot divide in suspension culture. Traction-mediated cytokinesis is conserved in mammalian cells as well^15^.

Cells are consistently subjected to wounding by physical or chemical damages from the external environment. In our bodies, the stretch and contraction of muscle tissue frequently injure the cell membrane^16,17^. *Dictyostelium* cells inhabit the surface of the soil, and may also be frequently wounded in severe environments. The cell membrane functions as a barrier between the extracellular and intracellular spaces. Wounded cell membrane loses its barrier function, resulting in an influx of undesirable substances such as Ca^2+^ ions into the cell as well as loss of cytoplasm. However, cells can repair the wounds on the cell membrane. Genetically errors in cell membrane repair may cause diseases, such as muscular dystrophy^16^. Therefore, wound repair is a physiologically essential process in living cells.

In large cells such as *Xenopus* eggs and *Drosophila* embryos, an actomyosin ring similar to the contractile ring in dividing cells surrounds the wound site. Its constriction facilitates the closure of the wound^18,19^. However, in smaller cells such as yeast cells, *Dictyostelium* cells, and cultured animal cells, actin alone transiently accumulates at the wound^20–22^. Under a deficiency of actin polymerization, wounds do not heal^23,24^. However, myosin II is not essential for the wound repair process in *Dictyostelium* cells^25^.

We previously showed that a wound modulates the polarity of cell migration^26^. When a migrating *Dictyostelium* cell is wounded at its anterior region, the cell retracts its anterior pseudopods, extends a new pseudopod at the posterior region, and migrates in the opposite direction; however, when wounded in the posterior part, the cell does not change its polarity but moves away from the wounded site with an increasing speed. These “escape” behaviors are independent of myosin II because myosin-null cells also move away from wound site in a similar manner^26^.

Here, we examined the effects of wounds at the cell membrane in dividing *Dictyostelium* cells using a laserporation technique—originally invented by us for introducing external substances into cells^27^. When the cell membrane at the cleavage furrow was wounded, myosin II transiently disappeared. Interestingly, the furrow constriction was significantly accelerated. However, when the cell membrane outside the furrow was wounded, the furrow constriction did not accelerate. Instead, the wounded-daughter half became smaller and the unwounded half larger, resulting in an asymmetrical cell division. When cells in the anaphase were wounded, they changed their polarity of division. These wound experiments on the dividing cells give new insights into the mechanism of cytokinesis and cell polarity establishment.

## Results

### Wound accelerates constriction of the cleavage furrow

**Figure 1:**
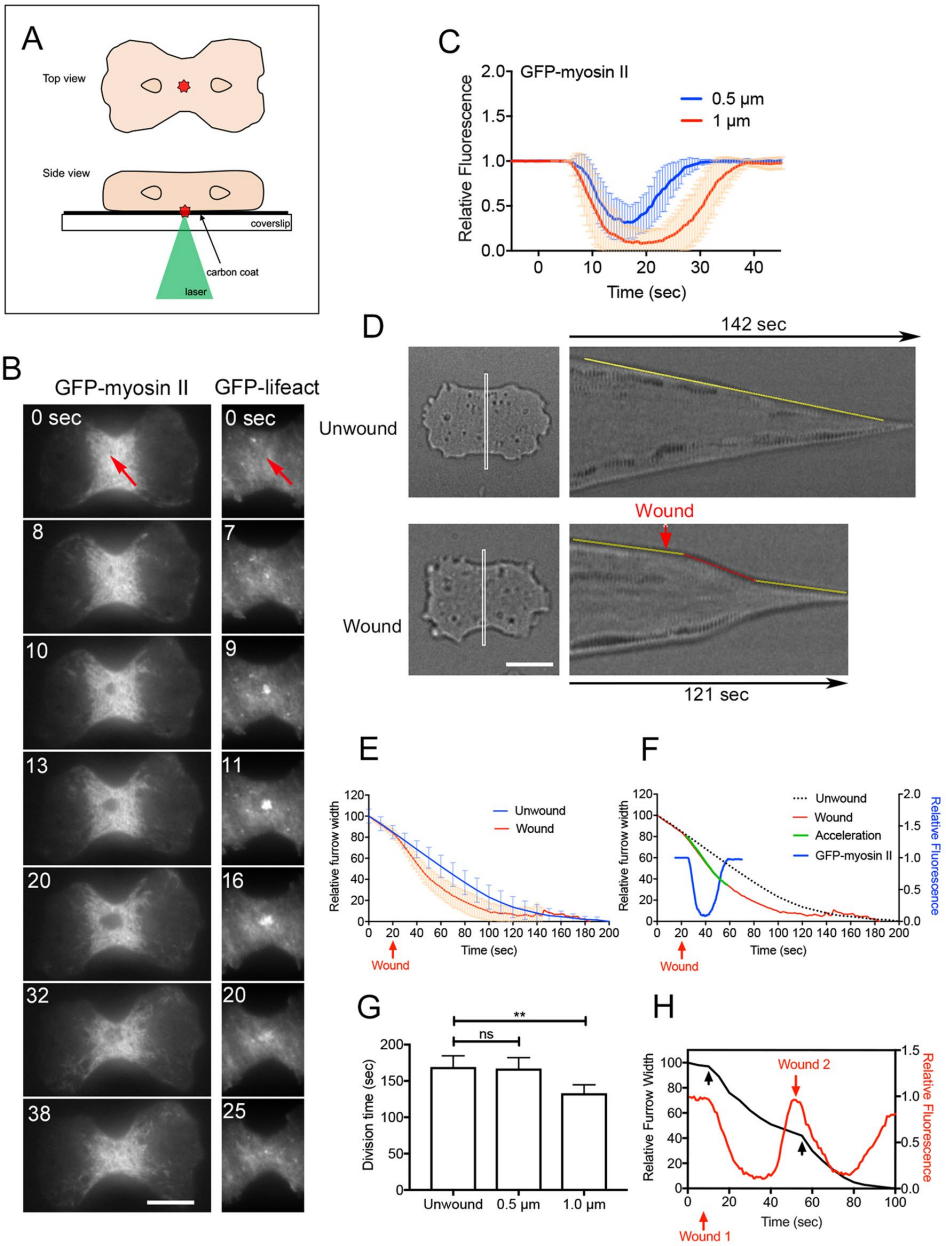
Wound accelerates constriction of the cleavage furrow. (**A**) Schema for the laserporation method: to make a wound in the cell membrane, cells were placed on a carbon-coated coverslip, and a laser beam was focused on a small local spot beneath a single cell under a TIRF microscope. The wound was set at a diameter of 0.5 or 1 μm. (**B**) Right panels show a typical time course of fluorescence images of a dividing cell expressing GFP-myosin II when the furrow membrane was wounded. Left panels show a typical time course of fluorescence images of the furrow region of a dividing cell expressing GFP-lifeact when the furrow membrane was wounded. Bar, 10 µm. (**C**) Time courses of fluorescence intensities of GFP-myosin II at the wound site when the furrow membrane was wounded with different sizes (0.5 and 1 µm in diameter) (n = 25 for each). (**D**) Typical kymographs of the white rectangles drawn in left panels. Upper panels show the kymograph without wounding as a control. The constriction rate was almost constant (yellow line). Lower panels show the kymograph of a wounded cell. The constriction was accelerated with a constant rate for about 40 s about 9 s after wounding (red line), and thereafter the constriction rate returned to the normal level (yellow line). Bar, 10 µm. (**E**) Time courses of the width of the furrow with and without wounding, respectively. (n = 25 for each). (**F**) Time course of myosin II dynamics (blue) is plotted in graph (**E**). The acceleration of furrowing is depicted in green. (**G**) Duration of cell division (from the initiation of furrowing to the final abscission) with wounding (0.5 and 1.0 µm) and without wounding. Data are presented as the mean ± SD. **P < 0.0001, ns, not significant; P > 0.05 (n = 25 for each). (**H**) A typical time course of relative fluorescence intensities of myosin II and the furrow width when the laser was applied two times (red arrows). The furrowing was accelerated each time (black arrows).

We used laserporation, our previously invented technique^27^, to examine the effects of wounds at the cleavage furrow in dividing cells. After the cells were placed on a coverslip coated with carbon by vapor deposition, a laser beam was focused on a small area in the furrow membrane under a TIRF microscope. The laser beam's energy absorbed by the carbon made a small pore in the cell membrane attached to the carbon coat (Fig. 1A). This method does not disrupt the actin cortex but makes a pore in the cell membrane^28^.

The left panels in Fig. 1B show a typical time course of fluorescence images of dividing cells expressing GFP-myosin II when the furrow membrane was wounded (arrow, wound size: 1 µm). In order to attach the furrow membrane to the substrate for TIRF microscopy, the cells were slightly pressed by using an agar overlay. Under this condition, individual filaments of myosin II were observed to accumulate at the cleavage furrow, and cells normally divided, as described previously^29–31^. When the furrow membrane was wounded, myosin II filaments began to disappear from the wound site at about 6 s, the dark spot expanded, and finally, the GFP-myosin II filaments reappeared. The right panels in Fig. 1B show a typical time course of fluorescence images of dividing cells expressing GFP-lifeact, a marker of actin filaments when the furrow membrane was wounded. Similar to our previous observations^25^, actin transiently accumulated between 2.5 and 26 s, with a peak at 10 s, after wounding.

Figure 1C shows the time courses of fluorescence intensities of GFP-myosin II at the wound site when the cells were wounded with different wound sizes (0.5 and 1 µm in diameter); larger responses were observed with larger wound sizes. We expected that the disappearance of myosin II would delay constriction or cancel cytokinesis. Figure 1D, E show kymographs of the white rectangles at the furrows and the time courses of the constriction (width of the furrow), respectively. Contrary to our expectation, the constriction was accelerated upon wounding.

The furrowing acceleration did not occur when the cells were wounded before the initiation of furrowing (n = 115), although the cortical myosin II transiently disappeared. The constriction was accelerated with a constant rate (0.11 ± 0.02 µm/s) for about 40 s (40.0 ± 5.0, n = 25) about 9 s (8.8 ± 2.2, n = 25) after wounding, and thereafter the constriction rate returned to the normal level (0.08 ± 0.02 µm/s). Figure 1F shows a comparison between the time courses of myosin II dynamics (blue) and furrow width (red), suggesting that furrowing was accelerated during myosin II disappearance. Figure 1G shows the duration of cell division (from the initiation of furrowing to the final division) with wounding (0.5 and 1.0 µm) and without wounding (n = 25 for each), suggesting that cell division is significantly accelerated by a wound larger than 1 µm. Figure 1H shows a typical time course of fluorescence intensities of myosin II and the furrow width when the laser was applied to the furrow two times (red arrows), suggesting that the furrowing was accelerated each time (black arrows); the wound-induced acceleration occurs at different timepoints of furrowing.

### Neither myosin II nor cortexillin is required for the furrowing acceleration

**Figure 2:**
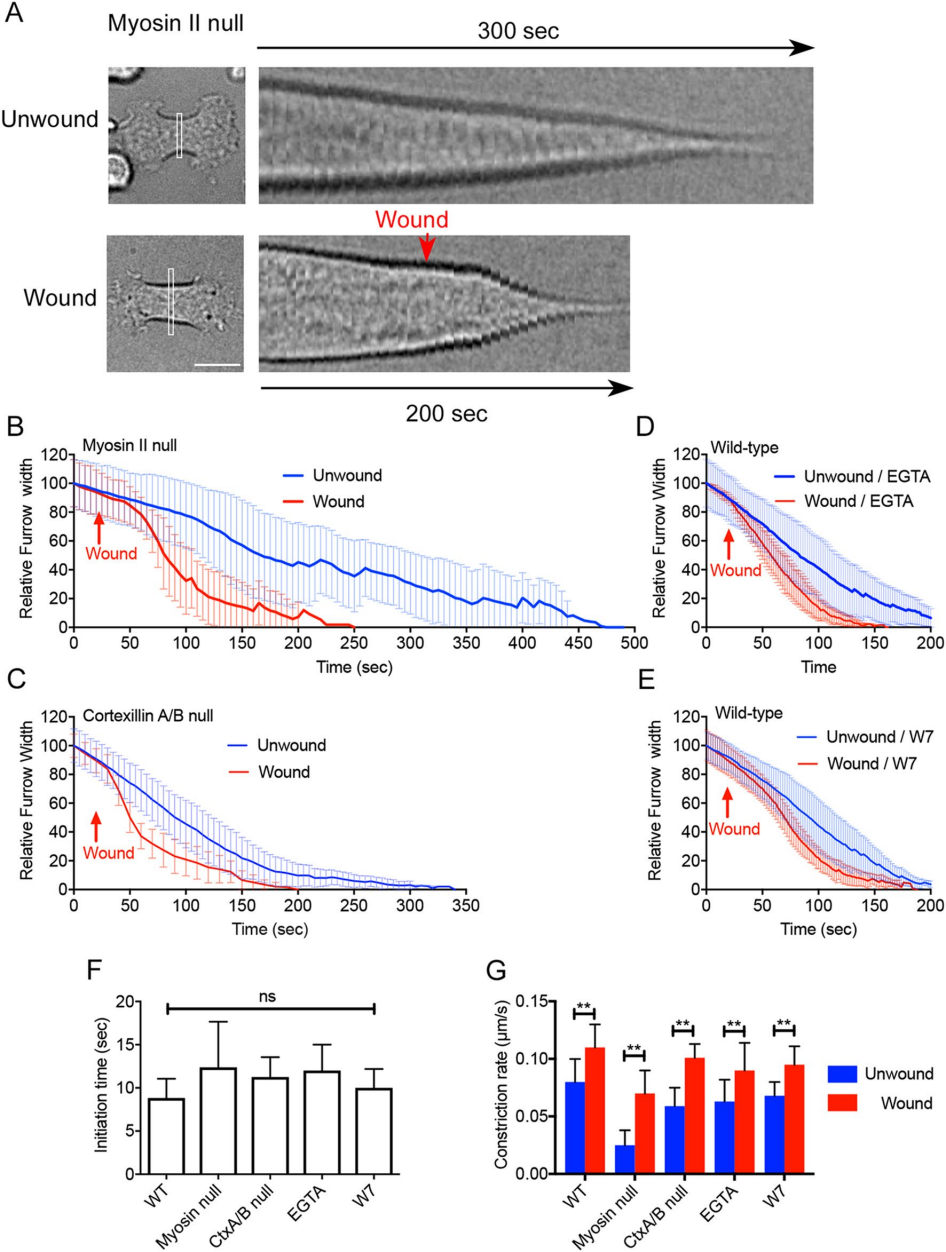
Wound repair is not required for the acceleration of furrow constriction. (**A**) Typical kymographs of the furrow width in myosin II-null cells with and without wounding. Bar, 10 µm. (**B**) Time courses of the furrow width in myosin II-null cells with and without wounding, respectively (n = 25 for each). (**C**) Time courses of the furrow constriction of dividing cortexillin A and B double-null cells with and without wounding, respectively (n = 25 for each). (**D**) Time courses of the furrow constriction of dividing wild-type cells in the presence of 10 mM EGTA with and without wounding, respectively (n = 25 for each). (**E**) Time course of the furrow constriction of dividing wild-type cells in the presence of W7 with and without wounding, respectively (n = 25 for each). (**F**) Initiation times of the acceleration after wounding in wild-type (WT) cells, myosin II null cells, cortexillin A/B (CtxA/B) null cells, wild-type cells in the presence of EGTA, and wild-type cells in the presence of W7, respectively. Data are presented as the mean ± SD. ns, not significant; P > 0.05 (n = 25 for each). (**G**) Constriction rates of furrowing in wild-type (WT) cells, myosin II null cells, cortexillin A/B (CtxA/B) null cells, wild-type cells in the presence of EGTA, and wild-type cells in the presence of W7 with and without wounding, respectively. The constriction rates were calculated using linear and the steepest parts of the graphs and kymographs. Data are presented as the mean ± SD. **P < 0.0001 (n = 25 for each).

To examine whether myosin II is required to accelerate furrow constriction, the furrow membrane in myosin II heavy chain-null cells (hereafter, myosin II-null cells) was wounded. Figure 2A,B show typical kymographs of the furrow width and the time courses of the constriction with and without the wound, respectively. Myosin II-null cells took longer to divide with a more significant deviation than wild-type cells^32^. Nevertheless, the furrow constriction was accelerated upon wounding, suggesting that myosin II is not required for wound-induced acceleration.

Cortexillin is a member of the alpha-actinin/spectrin superfamily, which localizes at the cleavage furrow and is required for its constriction^33–36^. We previously showed that cortexillin transiently disappears after wounding similar to myosin II^25^. Figure 2C shows the time course of furrow constriction in dividing cortexillin A and B double-null cells (Cortexillin A/B-null). Supplementary Fig. S1A shows typical kymographs of the furrow width with and without wounding. Furrow constriction was accelerated upon wounding, indicating that cortexillins A and B are not required for the acceleration.

### Wound repair is not required for the acceleration of furrow constriction

We have previously shown that the influx of Ca^2+^ from the external medium is essential for wound repair. The influx of Ca^2+^ triggers the accumulation of actin filaments and the disappearance of myosin II at the wound site. In the presence of EGTA, a Ca^2+^ chelator, in the external medium, neither actin accumulation nor disappearance of myosin II is observed^24,25^. Figure 2D shows the time courses of the constriction of the furrow of dividing wild-type cells in the presence of EGTA. Supplementary Fig. S1B shows typical kymographs of the furrow width with and without wounding. The furrow constriction was almost normally accelerated compared to that in the presence of Ca^2+^. However, most of the cells eventually ruptured after cytokinesis because the wound pores were not sealed in the absence of Ca^2+^. Therefore, the influx of Ca^2+^ is required for wound repair, but not for the acceleration of furrow constriction.

We have previously shown that calmodulin, a multifunctional Ca^2+^-binding messenger protein, accumulates at the wound site immediately after wounding. In addition, W7, an inhibitor of calmodulin, suppresses the calmodulin accumulation and the dynamics of actin and myosin II^24,25^. Figure 2E shows the time courses of the constriction of the furrow of dividing cells in the presence of W7. Supplementary Fig. S1C shows typical kymographs of the furrow width with and without wounding. The furrow constriction was also accelerated substantially in the presence of W7, suggesting that calmodulin is not required for the acceleration of furrow constriction. Figure 2F, G summarize the initiation times of the acceleration after wounding and the constriction rates of furrowing under the above conditions, respectively. In all examined conditions, the initiation times were not significantly different.

Because the influx of Ca^2+^ and the accumulation of calmodulin are not required for the acceleration of furrow constriction, it is plausible that a wound is required for the acceleration but wound repair is not required.

### Furrowing is not accelerated by wounds outside the cleavage furrow

**Figure 3:**
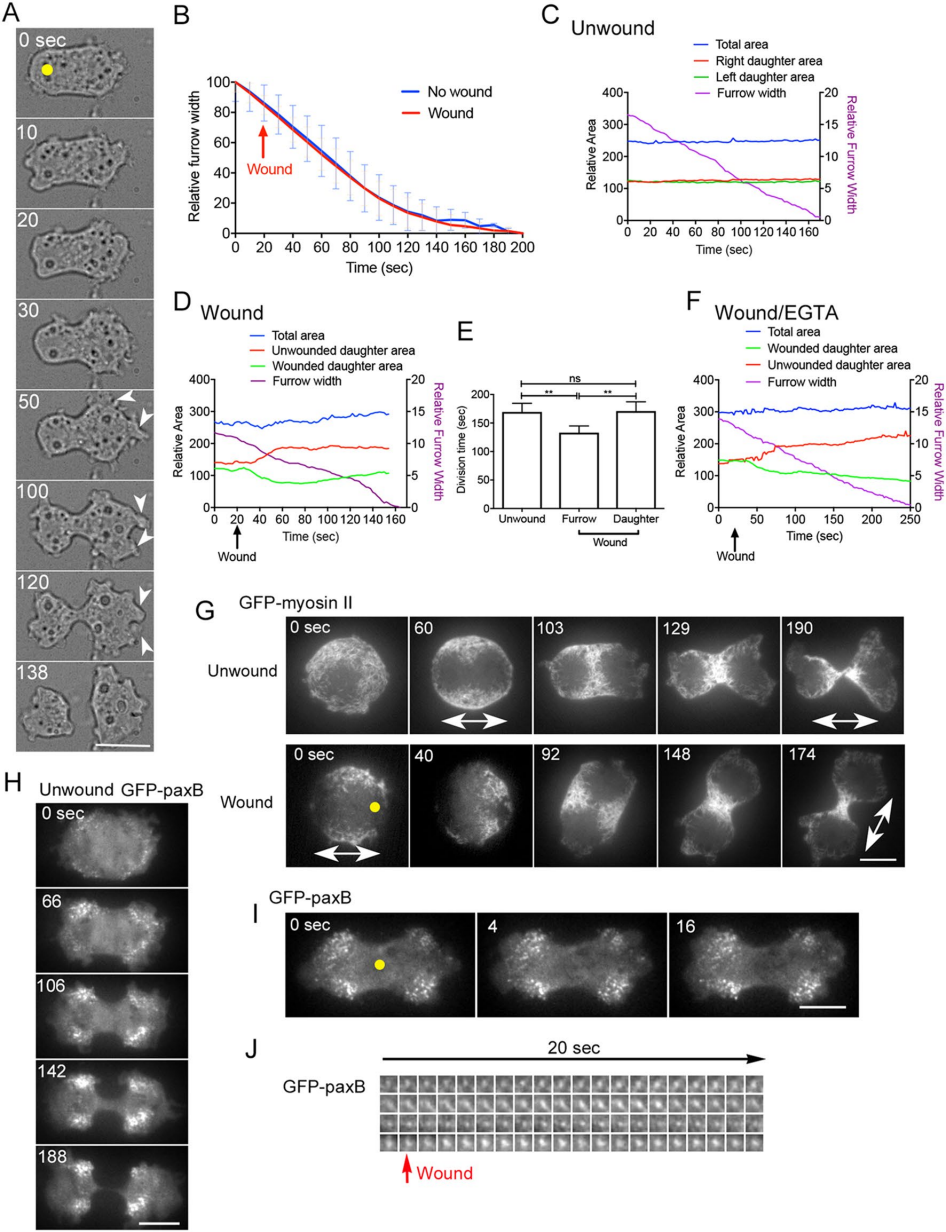
Wound outside the cleavage furrow induces asymmetrical cell division. (**A**) Typical time course of bright-field microscopy images after the daughter-cell membrane was wounded at the initiation time of furrowing (yellow dot). Arrow heads show polar pseudopods. Bar, 10 µm. (**B**) The time courses of the furrow width with and without wounding at the daughter-cell membrane (n = 25 for each). (**C,D**) Typical time courses of the areas of individual daughter halves (left and right), total area, and furrow width during cytokinesis with and without wounding. In (**D**), the cell was wounded at the left daughter cell membrane at 20 s. (**E**) Durations of cell division with wounding at furrow- and daughter-cell membrane and that without wounding, respectively. Data are presented as the mean ± SD. **P < 0.0001; ns, not significant; P > 0.05 (n = 25 for each). (**F**) Typical time courses of the areas of both daughter halves, total cell area, and furrow width with wounding at the daughter-cell membrane in the presence of EGTA. (**G**) Typical time courses of fluorescence microscopy images of dividing cells expressing GFP-myosin II with and without wounding. When wounded at the presumptive polar region (yellow dot) in anaphase cells, some cells (8/30) vastly changed their polarity (two-headed arrows). Bar, 10 µm. (**H**) Typical time course of TIRF microscopy images of a dividing cell expressing GFP-paxillin B without wounding. (**I**) Typical TIRF microscopy images of GFP-paxillin B upon wounding at the furrow membrane (yellow dot). (**J**) Time courses of 4 individual dots containing GFP-paxillin B upon wounding. Bars, 10 µm.

Next, we examined whether wounds at any location induce the acceleration of furrow constriction. Figure 3A, B show a typical time course of bright-field microscopy images and the time courses of the relative furrow width, respectively, when the cell membrane outside the cleavage furrow was wounded (yellow dot in Fig. 1A). Interestingly, the wounds did not accelerate furrow constriction; thus, the wound-induced acceleration is limited at the cleavage furrow.

### Wounds outside the cleavage furrow induce asymmetrical cell division

Figure 3C, D show the typical time courses of the areas of both daughter halves (red and green), total cell area (blue), and furrow width (purple) with and without wounding at the daughter-cell membrane, respectively. Interestingly, the size of the wounded-daughter half was reduced and that of the other half increased followed by the extension of multiple polar pseudopods (arrows), resulting in an asymmetrical cell division in all examined cells (n = 40). The duration of cell division did not change with wounding at the daughter-cell membrane (Fig. 3E). Figure 3F show typical time courses of the areas of both daughter halves, total cell area, and furrow width with wounding at the daughter-cell membrane in the presence of EGTA. The wound-induced asymmetrical cell division also occurred in the presence of EGTA (n = 20), suggesting that wound repair is also not essential. In addition, we never observed asymmetrical cell divisions when the furrow membrane was wounded.

Previously, we showed that local wounds induce changes in the polarity of migrating cells^26^. Herein, we examined whether the dividing cells changed the polarity of cell division (axis of cell division) upon local wounding. When the cell membrane of dividing cells in anaphase (after myosin II began to accumulate) was wounded at the presumptive polar region (yellow dot in Fig. 3F), some cells (8/30) vastly changed the axis of cell division, although most of the wounded cells (22/30) did not change the axis. However, wounding at the center of the cell never changed the cell polarity (n = 28). In addition, the cells in the cytokinesis stage (already furrowing) did not alter their polarity when any area such as the cell membrane of the daughter half or furrow membrane was wounded (n = 30).

### Wounding does not affect cell adhesions

Adhesion of cells to the substrate may affect furrow constriction and polarity of cell division^37,38^. When cells are detached from the substratum by non-adhesive coating or cultured in suspension, they fail to undergo cytokinesis^13,32,39^. The cyclic stretch of the elastic substrate changes the direction of cell migration in *Dictyostelium* cells perpendicular to the stretching direction^40^. Furthermore, we recently found that the cyclic stretch changes the axis of cell division parallel to the stretching axis, independent of myosin II. *Dictyostelium* cells adhere to the substratum with focal adhesions (actin foci) in a manner similar to many animal cells^41^. The wound may directly or indirectly affect focal adhesions to accelerate furrow constriction. To examine this possibility, cells expressing GFP-paxillin B, where the latter is a component of focal adhesions^42–44^, were wounded. Figure 3H shows a typical time course of TIRF microscopy images of a dividing cell expressing GFP-paxillin without wounding. Focal adhesions, including GFP-paxillin B, were observed as puncta-like structures, mainly localized at collar regions behind the polar pseudopods. Figure 3I shows a typical time course of TIRF microscopy images of GFP-paxillin B upon wounding at the furrow (yellow dot). Figure 3J shows time courses of fluorescence images of 4 individual dots upon wounding (arrow). The focal adhesions neither change their position nor disappear upon wounding. Therefore, wounding does not seem to affect cell adhesion to accelerate furrow constriction.

## Discussion

In this study, we showed the effects of wounds in the cell membrane during cell division. We found that the laser-induced wounds at the cleavage furrow accelerated furrow ingression and significantly reduced cell division time. Although furrowing was accelerated during myosin II disappearance, myosin II was not required for the acceleration because myosin II-null cells also showed similar acceleration. Calmodulin and influx of Ca^2+^ were not needed for acceleration, although they were necessary for wound repair. Therefore, the wound repair mechanism does not contribute to the acceleration. Furthermore, the wound in the cell membrane outside the furrow did not accelerate furrow constriction. Thus, the wound-induced acceleration was limited at the cleavage furrow, though the wound-induced actin and myosin II dynamics occurred at any location, including the furrow and daughter cortices^25^.

Several studies have examined the effect of laser ablation at the furrow in yeasts, nematode embryos, and animal cultured cells during cytokinesis, but these studies aimed at targeting the actomyosin contractile ring^45–48^. When a part of the contractile ring is disrupted by local laser ablation, it can be restored and continue to constrict. Interestingly, the furrow constriction is completed at the same time as controls, suggesting the furrow ingression is accelerated after the ablation in nematode embryo^46^. This acceleration seems similar to our observation, but the cell membrane is not disrupted. On the other hand, our laserporation method does not disrupt the actin cortex but only the cell membrane^28^. It is plausible that cells have robustness for cytokinesis in the face of perturbations and uncertainty by multiple mechanisms.

**Figure 4:**
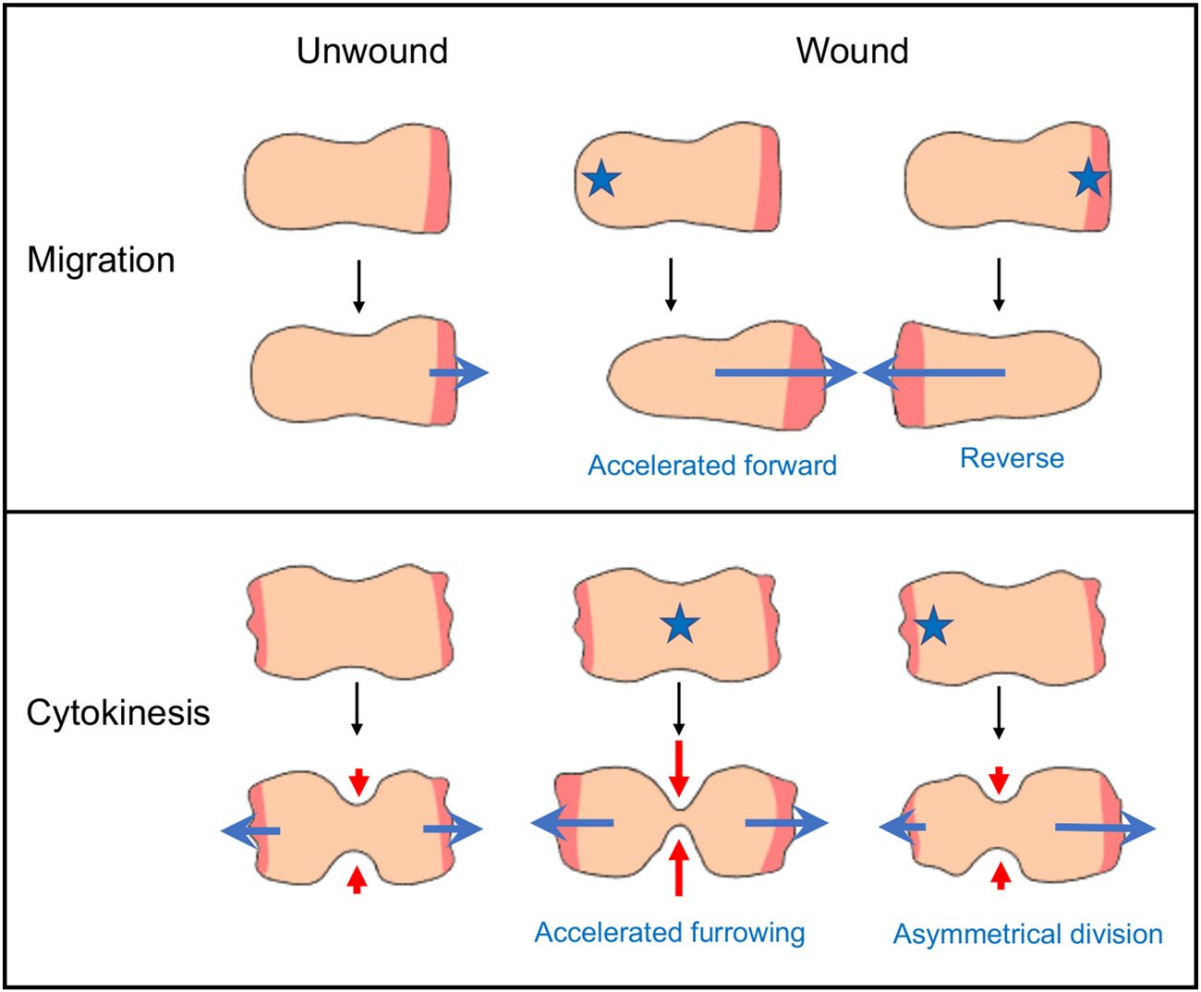
Summary of the behaviors of migrating and dividing cells upon wounding. When the posterior region of migrating cells is wounded, the cell migrates forward with an increased speed. When the anterior region of migrating cells is wounded, the cell migrates in opposite directions. When the furrow of dividing cells is wounded, the cell divides with an increased furrowing. When the daughter half of the dividing cells is wounded, the cell asymmetrically divides. Anterior and polar pseudopods are shown in red, wounds are shown by asterisks, the directions of cell movement are shown by blue arrows, and the directions of furrowing are shown by red arrows.

What is the physiological role of the acceleration of furrowing? As we previously discussed that the posterior wound-induced acceleration of cell migration and the anterior wound-induced change in the direction are ‘escape’ behaviors in wounded cells^26^. If dividing cells are considered two connected migrating cells moving in opposite directions, the molecular mechanism underlying the acceleration of furrowing may be the same as that of cell migration. Wounds at the cleavage furrow may force the cells to divide quickly in an emergency and those at the daughter cell membrane may force the other daughter half to enlarge to run away from the wound site, which results in asymmetrical cell division. Therefore, we concluded that dividing cells also show an escape behavior. Figure 4 summarizes the behaviors of migrating and dividing cells upon wounding.

Which force promotes the acceleration of furrowing? Myosin II-null and cortexillins-null cells also showed wound-induced acceleration, suggesting that these proteins are not responsible for the acceleration. Cytokinesis in wild-type *Dictyostelium* cells requires both contractile ring-dependent constriction and the traction forces of daughter halves migrating in opposite directions^13^. Myosin II-null cells can ingress cleavage furrows and divide only by the traction-mediated mechanism. This furrowing is considered to be passively generated due to the traction force (pulling force) of both daughter halves moving in opposite directions. However, the cortical tension (including Laplace pressure) may also contribute to the acceleration of furrowing^14,49^. Otherwise, the force may be generated by an actin-based mechanism, such as the use of an active force by cross-linking of actin filaments at the furrow^50,51^.

As the second candidate, cell-substratum adhesions may affect furrowing upon wounding because they are linked to actin structures and cell shape^37,52,53^. Traction force microscopy has shown that traction forces are mainly exerted at polar pseudopods in dividing halves^54^. If the adhesions around the furrow are reduced or those in polar regions are increased, the furrowing may be accelerated by the traction force of both daughter halves migrating in opposite directions. However, in the present study, localizations of paxillin B were not altered upon wounding (Fig. 3G). Other unknown adhesion mechanisms independent of paxillin B may contribute to the polarity of cell division. It was recently reported that discoidin I, a *N*-acetylgaractoseamine-binding lectin, contributes to cell-substrate adhesion and regulates contractile kits for cytokinesis^55^. We need to examine the traction force exerted on the substratum in association with adhesions upon wounding in the future.

Thirdly, membrane tension may contribute to the observed cell behavior. We previously examined many intracellular diffusive signals and lipid signals, but none of the signals contribute to the wound-induced escape behavior in migrating cells^26^. Membrane tension (including Laplace pressure) has been proposed to regulate cell motility and the anterior–posterior axis by coordinating with the cytoskeleton^56–59^ and to regulate the shape of dividing cells^14,49^. The membrane tension at the anterior end is generally higher than at the posterior end in directionally migrating cells^60,61^. We previously observed a temporal expansion of the pore in the cell membrane upon wounding, indicating that the membrane tension locally decreased upon wounding^62^. Presumably, the local reduction of the membrane tension generates a gradient of tension over the entire cell, which induces actin assembly to extend pseudopods at the site with the highest tension (the farthest from the wounding site), as shown in Fig. 4. According to this concept, when the daughter half is wounded, actin accumulates to extend polar pseudopods of the opposite daughter half, resulting in the enlargement of this half. When the cleavage furrow is wounded, both polar pseudopods are extended, which accelerates the furrowing. The measurement of the membrane tension has been limited to the tethering of the membrane with optical trapping or aspiration with a micropipette, which may result in some mechanical response of the cells. Recently, a fluorescent probe to measure the membrane tension has been invented^63,64^. We plan to measure the membrane tension upon wounding using this probe in the future.

## Materials and methods

### Cell culture

*Dictyostelium discoideum* (AX2) and all mutant cells were cultured at 22 °C in a plastic dish containing HL5 medium (1.3% bacteriological peptone, 0.75% yeast extract, 85.5 mM d-glucose, 3.5 mM Na_2_HPO_4_, and 3.5 mM KH_2_PO_4_; pH 6.3)^30^. For the wound experiments, the cells were suspended in HL5 medium supplemented with 3 mM CaCl_2_.

### Plasmids and transformation

GFP-myosin II and GFP-paxillin B expression constructs used here have been described in previous studies^43,65^. These constructs were transformed into cells using electroporation or laserporation, as described previously^27,66^. The transformed cells were selected in HL5 medium in plastic dishes containing 10 μg/mL G418 (Wako Pure Chemical Corporation, Osaka, Japan).

### Chamber preparation

The surface of the coverslip of a glass-bottom chamber was coated with carbon via vapor deposition, as previously described^27,62^; the coating layer was approximately 20 nm thick. The surface of the coated coverslip was activated by plasma treatment to make the surface hydrophilic. The chamber was sterilized with 70% ethanol when necessary. The cells were placed on the surface of the coated coverslip and slightly compressed with agarose block (2%, dissolved in BSS containing 10 mM NaCl, 10 mM KCl, 3 mM CaCl_2_, and 3 mM MES; pH 6.3; 1 mm thick) to observe the ventral cell surface^67,68^.

### Wounding and microscopy

As previously described, the cells expressing GFP were observed under a total internal reflection fluorescence microscope (TIRF; based on the IX71 microscope, Olympus, Japan)^69^. Cells were wounded with a nanosecond-pulsed laser (FDSS532-Q, CryLas, Germany), and the wound size was set to 0.5 or 1 μm in diameter^62^. Time-lapse fluorescence images were acquired with a 40–100-ms exposure time at 500-ms intervals using a CCD camera (Orca ER, Hamamatsu Photonics, Japan). The time courses of fluorescence intensities were examined using the Image J software (http://rsbweb.nih.gov/ij). The fluorescence intensities were measured within circles (1 µm diameter) including the wound at the center and normalized by setting the value before wounding to 1 after background removal.

### Inhibitors

W7 hydrochloride (Funakoshi Co. Ltd., Tokyo, Japan) was dissolved in dimethyl sulfoxide (DMSO) to prepare a 10 mM stock solution. Cells were incubated with a final concentration of 20 μM W7 hydrochloride in the medium for 30 min before the wounding experiments. For the EGTA experiments, cells were suspended in HL5 medium containing 10 mM EGTA, placed on the surface of the carbon-carted coverslip, and slightly compressed with agarose block (2%, dissolved in the buffer containing 10 mM NaCl, 10 mM KCl, 10 mM EGTA, and 3 mM MES; pH 6.3; 1 mm thick).

### Statistical analysis

Statistical analysis and linear regression analysis were conducted using GraphPad Prism 8 (https://www.graphpad.com; GraphPad Software, Inc., San Diego, CA, USA). Data are presented as mean ± standard deviation (SD) and were analyzed using unpaired two-tailed Student’s *t*-test or one-way ANOVA with Tukey’s multiple comparison test.

## Supplementary Information

Supplementary Figure S1.

## Data Availability

All relevant data are available from the authors on reasonable request.
