# Supplementary material for: Effects of wounds in the cell membrane on cell division
Source: Sci Rep. 2023 Feb 2;13:1941. doi: 10.1038/s41598-023-28339-z (PMC9895069; doi:10.1038/s41598-023-28339-z)
Supplement: Supplementary file 1 — Supplementary Figure S1. [file 41598_2023_28339_MOESM1_ESM.pdf]

Effects of wounds in the cell membrane on cell division

Md. Istiaq Obaidi Tanvir and Shigehiko Yumura

Supplementary Figure S1

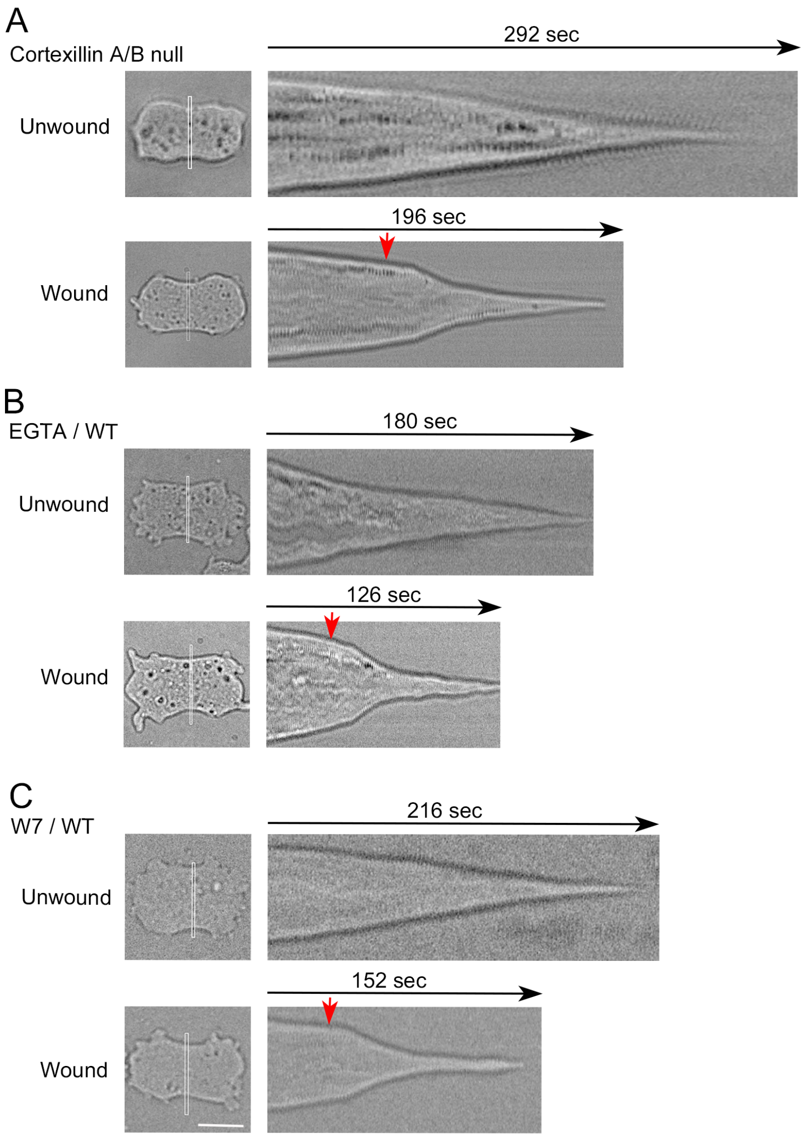

Supplementary Figure S1

(A) Typical kymographs of the furrow width in a cortexillin-null cell with and without wounding, respectively. (B) Typical kymographs of the furrow width in the presence of EGTA with and without wounding, respectively. (C) Typical kymographs of the furrow width in the presence of W7 with and without wounding, respectively. Red arrows show the time of wounding. Bar, 10 µm.
